# Rediscovery of *Lobonychiumpalpiplus* Roewer, 1938 (Opiliones, Laniatores, Epedanidae) in Sabah, Malaysia

**DOI:** 10.3897/zookeys.785.26389

**Published:** 2018-09-13

**Authors:** Chao Zhang, Jochen Martens

**Affiliations:** 1 The Key Laboratory of Invertebrate Systematics and Application, College of Life Sciences, Hebei University, Baoding, Hebei 071002, China Hebei University Hebei China; 2 Institut für Organismische und Molekulare Evolutionsbiologie, D-55099 Mainz, Germany Institut für Organismische und Molekulare Evolutionsbiologie Mainz Germany; 3 Senckenberg Research Institute, Frankfurt am Main, Germany Senckenberg Research Institute Frankfurt am Main Germany

**Keywords:** Arachnida, harvestmen, genitalia, functional morphology, taxonomy, Indonesia

## Abstract

*Lobonychiumpalpiplus* Roewer, 1938, originally reported from Indonesian Borneo, is redescribed based on the specimens from Malaysia. The genitalia of this species are described for the first time and a new genital terminology is proposed. The rediscovery expands the known distribution of the species to Malaysian Borneo.

## Introduction

The monotypic epedanid genus *Lobonychium* Roewer, 1938 was previously known from three specimens of the nominate species *L.palpiplus* Roewer, 1938, collected in the area of Pontianak (Kalimantan Barat, Indonesia, island of Borneo). The species is peculiar in having seven ventral and medial setiferous tubercles on the femur of the pedipalp and basal lobes on the claw of tarsi III and IV. The types are deposited in the Naturmuseum Senckenberg, Sektion Arachnologie, Frankfurt am Main, Germany. Except for the original description, the genus and species have not been mentioned in the literature during the past eighty years.

In February 2017, the senior author was able to re-examine the type specimens. Moreover, Malaysian Borneo, to the north of the type locality, was visited in October 2015 and May 2017 and several specimens (male and female) of *L.palpiplus* were collected. The newly discovered *Lobonychium* specimens are redescribed and illustrated.

The functional morphology of the male genitalia has been elaborated in some Laniatores families, e.g., Assamiidae Sørensen, 1884; Biantidae Thorell, 1889; Fissiphalliidae Martens, 1988; Phalangodidae Simon, 1879; Podoctidae Roewer, 1912; Stygnommatidae Roewer, 1923; Zalmoxidae Sørensen, 1886, etc. (Martens 1986, 1988; Ubick and Briggs 1992; Kury and Pérez-González 2002; Schwendinger 2006; Pérez-González 2007). In order to improve the morphological knowledge of Epedanidae Sørensen, 1886 we describe for the first time the expanded and unexpanded male genitalia.

## Materials and methods

Taxonomic methods follow the outline proposed by Acosta et al. (2007). The type material of *Lobonychium* is preserved in 70% denatured ethanol, and the specimens were examined under a Leica MZ16 at the Senckenberg Museum, Frankfurt, Germany (SMF). Non-type specimens were preserved in 75% ethanol, examined and drawn under a Leica M205A stereomicroscope. Photographs were taken using a Leica M205A stereomicroscope equipped with a DFC450 CCD. The male genitalia were placed first in hot lactic acid, then transferred to distilled water to expand the movable parts for observation (Schwendinger and Martens 2002). The terminology of genital structures follows Macías-Ordóñez et al. (2010), and the macrosetae terminology follows Kury and Villarreal (2015). Non-type material is deposited in the Museum of Hebei University, Baoding, China (MHBU). All measurements are given in mm.

## Taxonomy

### Epedanidae Sørensen, 1886

#### Epedaninae Sørensen, 1886

##### Lobonychium

Taxon classificationAnimaliaOpilionesEpedanidae

Roewer, 1938

Lobonychium Roewer, 1938: 125.

###### Type species:

*Lobonychiumpalpiplus* Roewer, 1938, by monotypy.

###### Etymology.

The name *Lobonychium* is derived from the Greek *λοβός*, meaning lobe of the ear, and the Latinized Greek *onyx* (genitive *onychos*), meaning claw.

###### Diagnosis.

Body, including ocularium, unarmed. Basichelicerite and tibia II elongate. Basichelicerite dorsally with conspicuous granules. Femur of pedipalp ventrally with a row of seven setiferous tubercles. Distitarsus I with two tarsomeres, distitarsus II with three. Double claws of tarsi III–IV with basal lobes, untoothed; no scopula. Ventral plate of penis forming a heart-shaped stereoscopic structure (ventral view, Fig. 28). Glans partially sunken into dorsally depressed portion of pars distalis (Fig. 26). Stylus with capsula interna sunken into capsula externa (Fig. 27).

###### Sexual dimorphism.

Tibia II in male distended at distal portion, but normal in female.

###### Remarks.

Within the subfamily Epedaninae, *Lobonychium* is morphologically similar to *Epedanidus* Roewer, 1943, *Metepedanulus* Roewer, 1913, and *Zepedanulus* Roewer, 1927 in having an unarmed body. Besides, *Lobonychium* and *Caletorellus* Roewer, 1938 have characteristic basal lobes or medial branches on double claws of tarsi III–IV (Roewer 1938: 125, 129, fig. 4 b–d). However, *Lobonychium* is noticeably distinct from these epedanids by the pedipalp femur, medially with a row of seven setiferous tubercles.

###### Distribution.

The type locality is at or near the city of Pontianak (Fig. 36 A), Indonesia. The new records are from Malaysia, at Trus Madi Mountain (Fig. 36 B) and Kalabakan (Fig. 36 C).

##### Lobonychium palpiplus

Taxon classificationAnimaliaOpilionesEpedanidae

Roewer, 1938

Figs 1–7 , 8–22 , 23–30 , 31–34 , 36

Lobonychium palpiplus Roewer, 1938: 125, fig. 44.

###### Type specimens.

Male lectotype (SMF-5376-1201), here designated, labeled: “*Lobonychiumpalpiplus*, male lectotype (SMF-5376-1201), designated by Chao Zhang [handwritten]”.

All types (lectotype and two paralectotypes) from Pontianak [00°01'S, 109°20'E], Borneo (West Kalimantan, Indonesia), deposited in the Senckenberg Museum Frankfurt, Germany, labeled: “Arachn. Coll. Roewer – Lfd. No. 5376, Opil: Epedaninae No. 16, *Lobonychiumpalpiplus* Rwr [abbreviation for Roewer], 2♂1♀, Borneo: Pontianak, Typus, Roewer det. 1935” (Fig. 35) (examined).

Additional material examined. 1♂ (MHBU-Opi-20151208m) and 2♀, (MHBU-Opi-20151208f, MHBU-Opi-2015120801f), Malaysia: Sabah, Trus Madi Mountain, about 1103 m alt. 05°25.637'N, 116°25.984'E, October 9, 2015, Z. Z. Gao leg.; 1♀, Malaysia: Sabah, Trus Madi Mountain, about 1081 m alt. 05°26.111'N, 116°27.237'E, October 10, 2015, Z. Z. Gao leg.; 1♀, Malaysia: Sabah, Trus Madi Mountain, about 760 m alt. 05°27.598'N, 116°26.936'E, October 12, 2015, Z. Z. Gao leg.; 1♀, Malaysia: Sabah, Kalabakan, about 330 m alt. 04°32.522'N, 117°10.020'E, October 18, 2015, Z. Z. Gao leg.; 2♀, Malaysia: Sabah, Trus Madi Mountain, about 1121 m alt. 05°26.529'N, 116°27.309'E, May 1, 2017, C. Jin leg.; 1♂1♀, Malaysia: Sabah, Trus Madi Mountain, about 1081 m alt. 05°26.111'N, 116°27.237'E, May 3, 2017, C. Jin leg.

###### Redescription.

**Figures 1–7.**
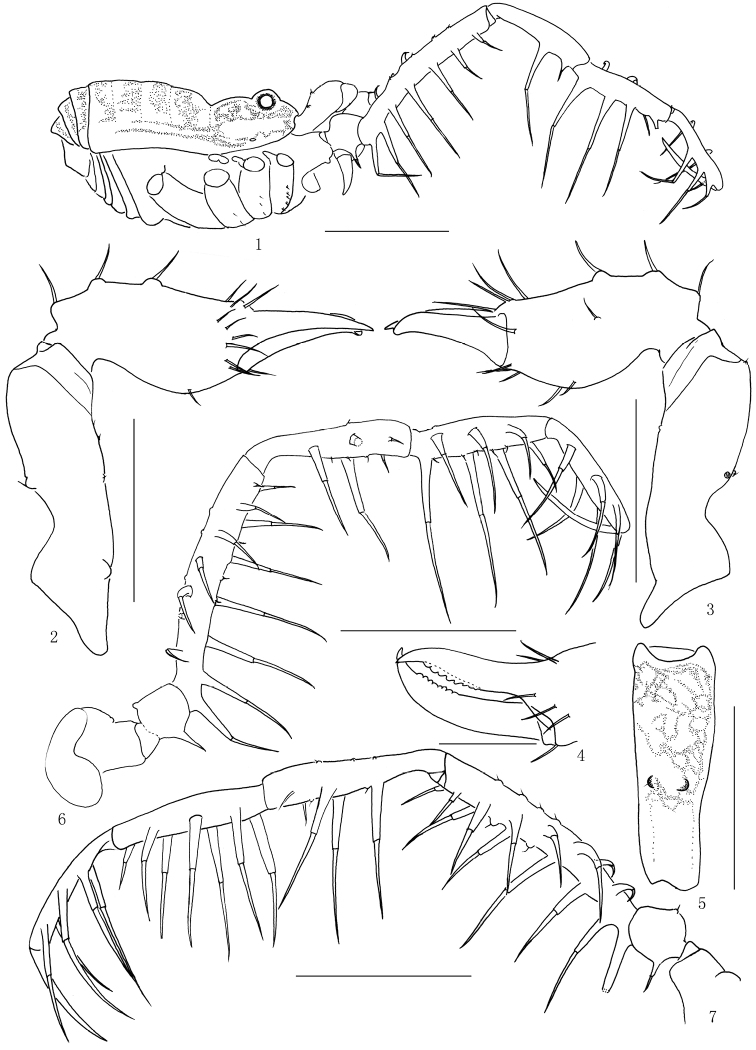
*Lobonychiumpalpiplus* Roewer, 1938, male (MHBU-Opi-20151208m) (**1–6**), female (MHBU-Opi-20151208f) (**7**) **1** Body, lateral view **2** Left chelicera, medial view **3** Same, ectal view **4** Cheliceral fingers, frontal view **5** Basal segment of left chelicera, dorsal view **6** Left pedipalp, medial view **7** Right pedipalp, medial view. Scale bars: 1mm (**1, 6–7**); 0.5 mm (**2–3, 5**); 0.25 mm (**4**).

**Figures 8–22.**
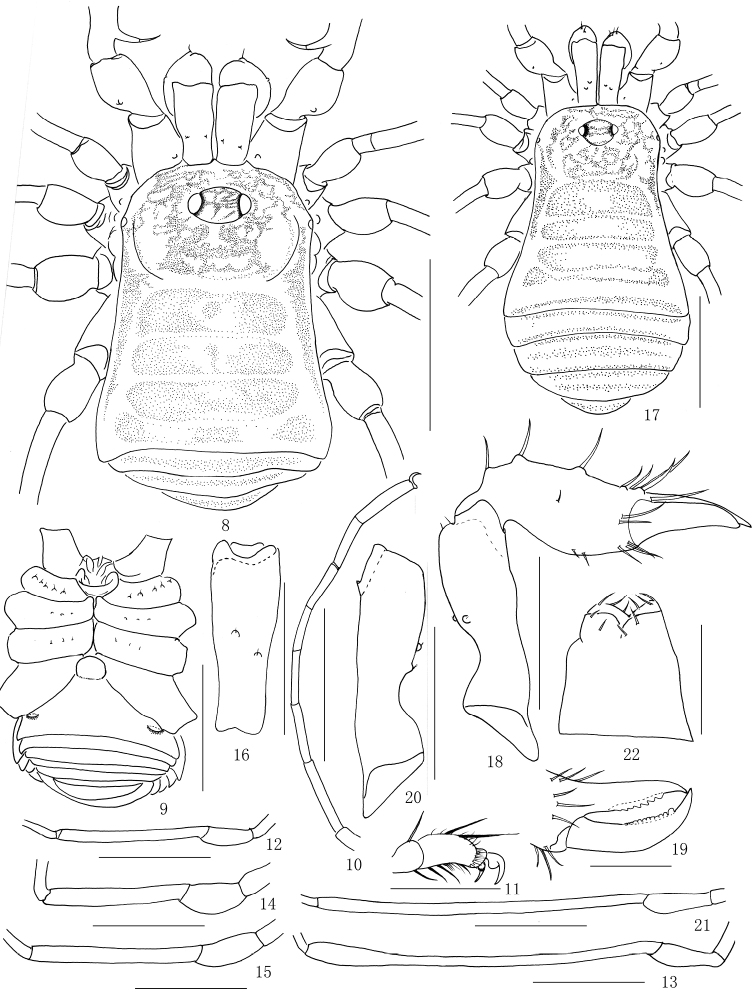
*Lobonychiumpalpiplus* Roewer, 1938, male (MHBU-Opi-20151208m) (**8–16**), female (MHBU-Opi-20151208f) (**17–22**) **8** Body, dorsal view **9** Same, ventral view **10** Right tarsus I, retrolateral view **11** Distal segment of right tarsus III, retrolateral view **12–15** Patellae and tibiae of left legs, prolateral view **12** Leg I **13** Leg II **14** Leg III **15** Leg IV **16** Basal segment of right chelicera, dorsal view **17** Body, dorsal view **18** Right chelicera, ectal view **19** Cheliceral fingers, frontal view **20** Basal segment of left chelicera, ectal view **21** Patella and tibia of left leg II, prolateral view **22** Ovipositor, ventral view. Scale bars: 1 mm (**8–9, 12–15, 17, 21**); 0.5 mm (**10, 16, 18, 20**); 0.25 mm (**11, 19, 22**).

Male (MHBU-Opi-20151208m) habitus as in Figs 1, 8–9, 31. Coloration (Fig. 31): entire body rusty yellow, with somewhat dark brown to blackish brown patches on dorsum; median area of carapace with dark brown reticulations; both lateral ridges of scutum with blackish brown stripes; opisthosomal region of scutum banded with a dark brown outline; a dark brown band across posterior margin of scutum; free tergites I–III each with a dark brown band; coxa with dark brown reticulations; free sternites with transverse dark brown band; chelicerae and pedipalp reticulated; trochanters of all legs pale yellow, femur, patella, tibia and metatarsus with black reticulations, tarsus lighter.

**Dorsum** (Figs 8, 31). Scutum elongate in appearance, both sides straight, nearly parallel, widest portion of body at scutal area IV, abdomen bluntly pointed posteriorly. Carapace unarmed on lateral portion of anterior margin. Surface of dorsum smooth. Ocularium low and oval, unarmed, removed from anterior border of scutum by 0.16 mm. Borders of opisthosomal scutum parallel to each other. Free tergites and anal operculum unarmed.

**Venter** (Fig. 9). Surface of coxa I tuberculated, antero-dorsally with a coarse tubercle, and a row of five tubercles on ventral surface. Coxa II with a row of small granules on ventral surface. Coxae III and IV nearly smooth aside from a row of small teeth on front and rear margins of coxa III. Genital operculum and free sternites with seta-tipped granules. Spiracles clearly visible.

**Chelicerae** (Figs 2–5, 16). Basichelicerite elongate, with a slight bulla and armed with two conspicuous granules at base of bulla, medial surface with a basal protuberance (Figs 2, 3, 5). Cheliceral hand with some greater seta-tipped tubercles. Fingers relatively short, inner edges toothed (Fig. 4): moveable finger and fixed finger with eight crested teeth, respectively.

**Pedipalpi** (Fig. 6). Coxa dorsally with one small tubercle near distal margin. Trochanter ventrally with one setiferous tubercle and dorsally with one small tubercle. Femur ventrally with a row of seven setiferous tubercles of even size and spirally arranged from base to distal end on medial side; dorsally with many low conical tubercles along entire length. Patella ventro-mesally with two long and one short setiferous tubercles, and ventro-ectally with one long and one short setiferous tubercles. Tibia ventro-mesally with three setiferous tubercles, and ventro-ectally with four setiferous tubercles. Tarsus with three setiferous tubercles on each side of ventral surface. Tarsal claw curved, approximately same length as tarsus.

**Legs** (Figs 10–15). Slender and long. All segments unarmed, nearly smooth. Femora I–IV not curved, almost straight. Tibia II distended at distal portion, conspicuously longer than tibiae I, III and IV (Figs 12–15). Distitarsus I with two (Fig. 10), distitarsus II with three tarsomeres. Distitarsi III–IV without scopula. Each claw of tarsi III–IV with one basal lobe nearly circular in shape (Fig. 11). Tarsal formula (I–IV): 7/19/7/7.

**Figures 23–30.**
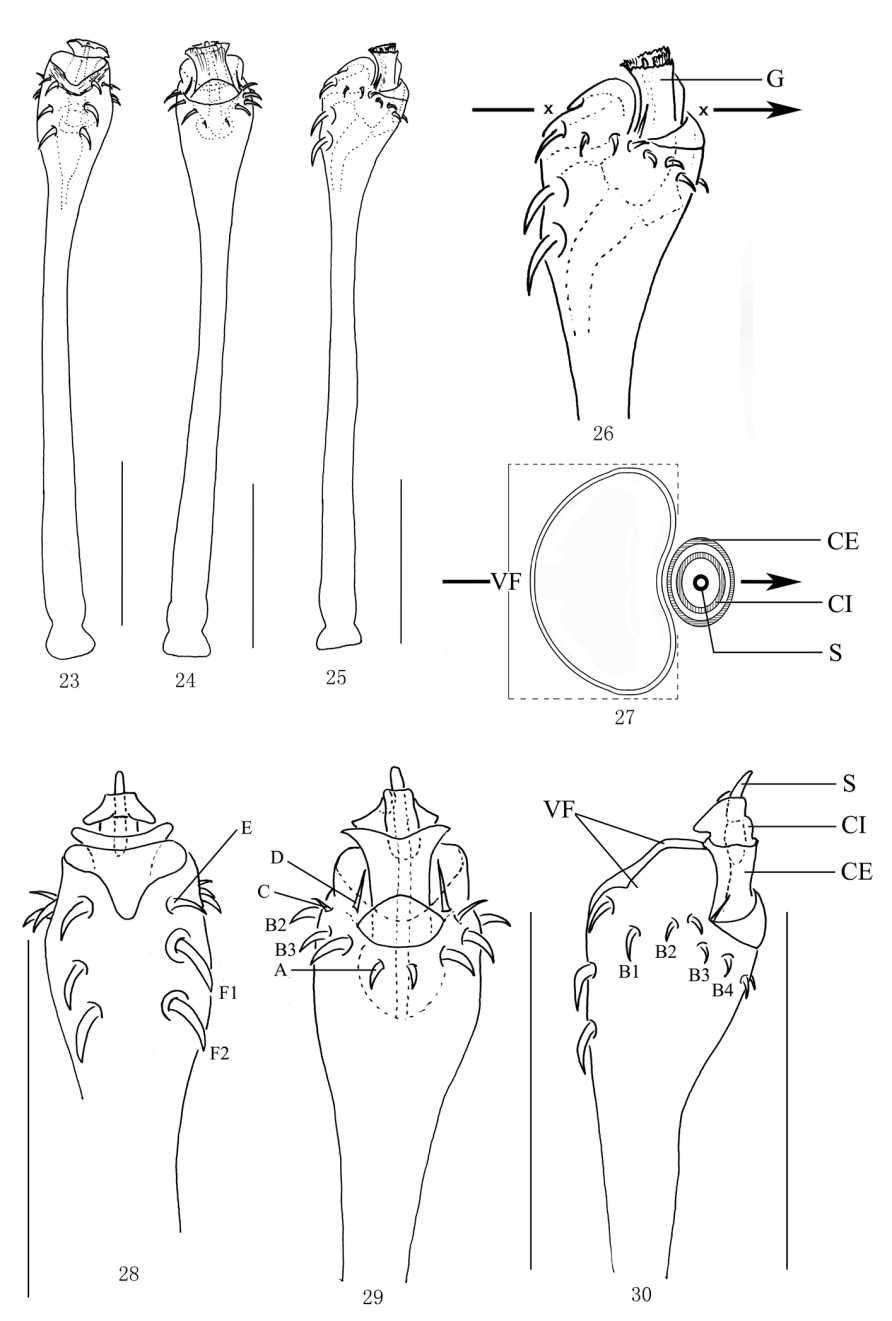
*Lobonychiumpalpiplus* Roewer, 1938 (MHBU-Opi-20151208m) **23** Penis, ventral view **24** Same, dorsal view **25** Same, lateral view **26** Distal part of penis, lateral view **27** cross-section through glans and truncus at the marked point **28** Distal part of penis (expanded), ventral view **29** Same, dorsal view **30** Same, lateral view. Abbreviations: **CE** capsula externa (follis) **CI** capsula interna **G** glans **S** stylus **VF** ventral frame. Scale bars: 0.25 mm.

**Penis** (Figs 23–30). Shaft slender, nearly parallel-sided, then distended towards apical portion (pars distalis). Ventral plate inflated, hollow on inner side, forming a heart-shaped stereoscopic ventral frame (ventral view, Fig. 28) (Figs 27, 30). Glans partially sunken into dorsal depressed portion of pars distalis (Fig. 26). Prior to inflation of capsula externa (follis) (Fig. 30), stylus with capsula interna sunken into capsula externa (Fig. 27). Capsula externa dorsally and ventrally extended at distal end. Everted capsula interna with a bifurcate ventral lobe distally. Stylus finger-shaped. Spination symmetrical. One pair of setae A, C, D, and E. Two pairs of setae F. Four pairs of setae B (Figs 28–30).

**Female** (MHBU-Opi-20151208f) (Figs 7, 17–22, 32–34). Generally similar to male except abdomen slightly wider than in male (Figs 17, 32). Tibia II conspicuously longer than tibiae I, III and IV, but inconspicuously distended distally (Fig. 21). Inner edges of finger of chelicera toothed (Fig. 19): moveable finger with 12 teeth; fixed finger with seven teeth. Tarsal formula (I–IV): 7/22/7/7.

**Figures 31–34.**
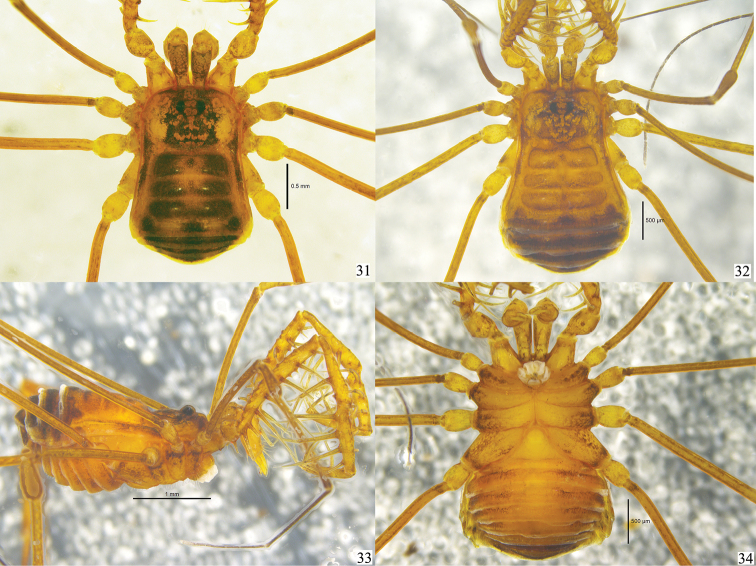
*Lobonychiumpalpiplus* Roewer, 1938 Photographs of male (MHBU-Opi-20151208m) and female (MHBU-Opi-20151208f) **31** Male body and parts of appendages, dorsal view **32** Female body and parts of appendages, dorsal view **33** Same, lateral view **34** Same, ventral view. Scale bars: 1 mm (Fig. 33); 0.5 mm (Figs 31–32, 34).

**Ovipositor** (Fig. 22). Ventral surface with four setae and dorsal surface with six setae.

###### Measurements.

Male (female): body 1.89 (2.74) long, 1.36 (1.64) wide at widest portion, scutum 1.69 (1.76) long. Ocularium 0.19 (0.20) long, 0.37 (0.35) wide. Proximal article of chelicera 0.69 (0.62) long, 0.24 (0.23) wide; second 0.89 (0.98) long, 0.26 (0.26) wide; distal 0.40 (0.44) long, 0.09 (0.08) wide. Pedipalp claw 0.64 (0.63) long. Penis 0.88 long. Measurements of pedipalp and legs as in Tables 1, 2.

**Table 1.** Pedipalp and leg measurements (mm) of *Lobonychiumpalpiplus* Roewer, 1938, male, length/width.

	Trochanter	Femur	Patella	Tibia	Metatarsus	Tarsus	Total
Pedipalp	0.32/0.28	1.47/0.20	0.97/0.19	0.77/0.18		0.71/0.14	4.24
Leg I	0.35/0.18	1.72/0.14	0.46/0.18	1.38/0.11	2.21/0.05	1.33/0.03	7.45
Leg II	0.36/0.18	3.34/0.10	0.66/0.18	2.99/0.17	3.75/0.05	2.80/0.04	13.90
Leg III	0.37/0.24	2.20/0.15	0.64/0.25	1.23/0.17	2.63/0.10	1.24/0.07	8.31
Leg IV	0.37/0.24	2.90/0.15	0.61/0.25	1.52/0.17	3.56/0.10	1.44/0.07	10.40

**Table 2.** Pedipalp and leg measurements (mm) of *Lobonychiumpalpiplus* Roewer, 1938, female, length/width.

	Trochanter	Femur	Patella	Tibia	Metatarsus	Tarsus	Total
Pedipalp	0.45/0.31	1.49/0.18	1.10/0.22	0.91/0.19		0.89/0.12	4.84
Leg I	0.37/0.19	1.62/0.10	0.48/0.18	1.36/0.10	2.20/0.06	1.34/0.04	7.37
Leg II	0.39/0.20	3.18/0.10	0.62/0.18	3.01/0.10	3.72/0.06	2.73/0.04	13.65
Leg III	0.42/0.26	2.13/0.12	0.54/0.26	1.28/0.16	2.54/0.09	1.10/0.06	8.01
Leg IV	0.42/0.26	2.88/0.12	0.54/0.26	1.61/0.16	3.41/0.09	1.43/0.06	10.29

###### Habitat.

The specimens were collected by leaf litter sieving in dark moist places under dense forest canopy.

###### Variation.

The collection examined contains ten specimens, two males and eight females. The male (MHBU-Opi-20151208m) and the female (MHBU-Opi-20151208f) described here are asymmetrical in the position of granules on the left (Figs 5, 20) and right (Figs 16, 18) basichelicerites of chelicerae. Another male body 1.96 long, 1.21 wide at the widest portion, scutum 1.68 long. Size range of other females (n=7) as follows minimum (maximum in parentheses): body 2.20 (2.70) long, 1.59 (1.76) wide at the widest portion, scutum 1.72 (1.89) long. The variations in the number of segments in the tarsus are shown in Table 3.

**Table 3.** Numbers of tarsomeres of on legs of *Lobonychiumpalpiplus* Roewer, 1938. L left, R right tarsus. Only one number is given when the specimen is symmetrical.

Specimen number (n=10)	Sex	Leg I	Leg II	Leg III	Leg IV
1	male	7	L19, R 21	7	7
2	female	7	L19, R 20	7	7
3, 4	male and female	7	19	7	7
5, 6	females	7	22	7	7
7, 8	females	7	18	7	7
9	female	7	L18, R 21	6	7
10	female	7	21	7	7

###### Distribution.

Indonesia (Pontianak), Malaysia (Trus Madi Mountain, Kalabakan).

###### Remarks.

The three type specimens have not been dissected and are in good condition, with all appendages attached. The original description of the types by Roewer (1938) corresponds more-or-less to the morphology of the type specimens except for few minor characters, e.g., the male tibia II is distended at its distal portion, the presence of one short setiferous tubercle ventro-mesally on the pedipalpal patella, the minimum numbers of the tarsomeres II and III are 18 and 6, respectively, and the smaller male body (1.89–1.96).

Additionally, the localities of the new records from Malaysia are at most about 120 km apart. The distance between the recorded type locality (Indonesian part of Borneo) and the new localities (Malaysian part) is nearly 1000 km (Fig. 36).

**Figure 35.**
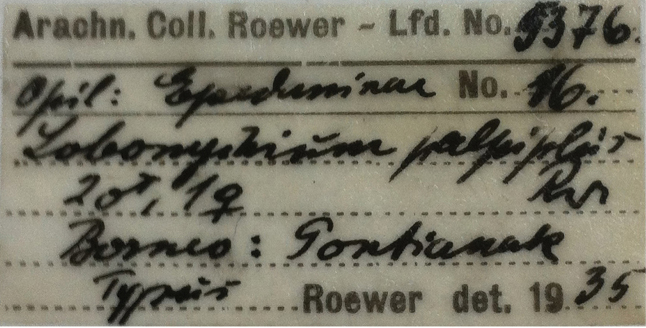
Original label with Roewer’s handwriting of the type series of *Lobonychiumpalpiplus* Roewer, 1938.

**Figure 36.**
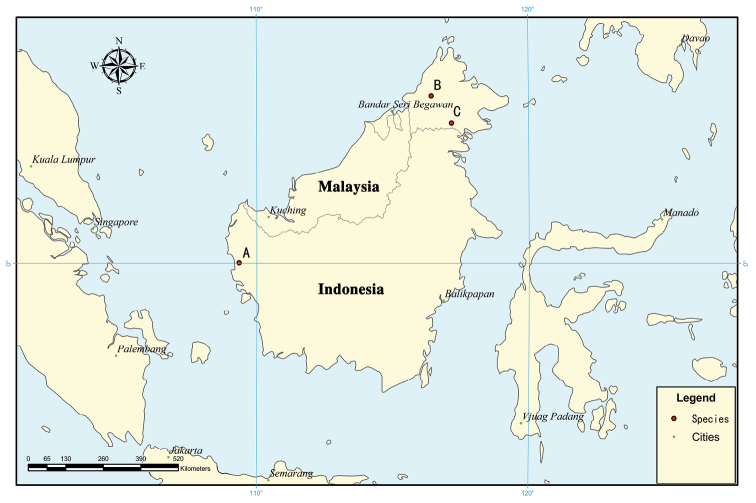
Distribution of *Lobonychiumpalpiplus* Roewer, 1938 in Borneo. **A** type locality **B** Trus Madi Mountain **C** Kalabakan.

According to the drawings presented by Suzuki (1969: 30, fig. 19 E; 1977: 19, fig. 6 F–G; 1981: 268, fig. 1B–C) the male genital morphology of Epedanidae seems to be quite homogeneous and little functional variation has been documented to date.

## Supplementary Material

XML Treatment for Lobonychium

XML Treatment for Lobonychium palpiplus
